# The methylation status of the chemerin promoter region located from − 252 to + 258 bp regulates constitutive but not acute-phase cytokine-inducible chemerin expression levels

**DOI:** 10.1038/s41598-020-70625-7

**Published:** 2020-08-13

**Authors:** Kamila Kwiecien, Piotr Brzoza, Maciej Bak, Pawel Majewski, Izabella Skulimowska, Kamil Bednarczyk, Joanna Cichy, Mateusz Kwitniewski

**Affiliations:** 1 Department of Immunology, Faculty of Biochemistry, Biophysics and Biotechnology, Jagiellonian University, 30-387 Krakow, Poland; 2 Swiss Institute of Bioinformatics, Biozentrum, University of Basel, 4056 Basel, Switzerland

**Keywords:** Interleukins, Oncostatin M, DNA methylation

## Abstract

Chemerin is a chemoattractant protein with adipokine properties encoded by the retinoic acid receptor responder 2 (*RARRES2*) gene. It has gained more attention in the past few years due to its multilevel impact on metabolism and immune responses. However, mechanisms controlling the constitutive and regulated expression of *RARRES2* in a variety of cell types remain obscure. To our knowledge, this report is the first to show that DNA methylation plays an important role in the cell-specific expression of *RARRES2* in adipocytes, hepatocytes, and B lymphocytes. Using luciferase reporter assays, we determined the proximal fragment of the *RARRES2* gene promoter, located from − 252 to + 258 bp, to be a key regulator of transcription. Moreover, we showed that chemerin expression is regulated in murine adipocytes by acute-phase cytokines, interleukin 1β and oncostatin M. In contrast with adipocytes, these cytokines exerted a weak, if any, response in mouse hepatocytes, suggesting that the effects of IL-1β and OSM on chemerin expression is specific to fat tissue. Together, our findings highlight previously uncharacterized mediators and mechanisms that control chemerin expression.

## Introduction

Chemerin is a small (18 kDa) multifunctional protein capable of regulating different biological processes, including immune cell migration, adipogenesis, osteoblastogenesis, angiogenesis, myogenesis, and glucose homeostasis^1^. Moreover, it shows broad-spectrum antimicrobial activity in both human and mouse epidermis, suggesting it plays a role in maintaining skin-barrier homeostasis^2,3^. Chemerin-induced signaling is mediated predominantly through chemokine-like receptor 1 (CMKLR1), which is expressed by many cells including plasmacytoid dendritic cells (pDCs), macrophages, natural killer (NK) cells, adipocytes, hepatocytes, and keratinocytes^2,4–9^. Chemerin is secreted as pro-chemerin and circulates in plasma as an inactive precursor protein (Chem163S) that can subsequently be activated through posttranslational carboxyl-terminal processing by a variety of proteinases^10,11^.

The gene encoding chemerin is known as retinoic acid receptor responder 2 (*RARRES2*)^12^, or as tazarotene-induced gene 2 (*TIG2*) given it was first discovered in tazarotene-treated psoriatic skin lesions^13,14^. Liver and adipose tissue are reported to be the major sites of chemerin production; nonetheless, *RARRES2* mRNA is detectable in many other tissues, including the adrenal glands, ovaries, pancreas, lungs, kidney, and skin^2,15^. Chemerin expression in these tissues may be constitutive and/or regulated^1^. It is likely that these pathways are controlled differently. For example, adipocytes and hepatocytes show high constitutive *RARRES2* mRNA levels^15^, whereas the chemerin transcript is not detectable in bone marrow or immune cells, such as monocytes or granulocytes^16^. So far, it has not been determined what controls the on/off “switch” of the chemerin expression in different cells.

Chemerin expression may be regulated by a variety of inflammatory and metabolic mediators in a manner dependent on cell type^17^. These factors can be broadly classified as (1) agonists of nuclear receptors (retinoids, vitamin D, glucocorticoids), (2) factors mainly associated with metabolic processes (e.g. fatty acids, insulin, glucose) and (3) immunomodulatory mediators (e.g. cytokines of acute or chronic inflammation and lipopolysaccharide (LPS)^1^. The molecular mechanisms underlying the regulated expression of chemerin are poorly understood. Analysis of the chemerin promoter has identified functional response elements for the peroxisome proliferator-activated receptor γ (PPARγ), farnesoid X receptor (FXR), and sterol regulatory element-binding protein 2 (SREBP2) in the mouse chemerin promoter^18–20^. These factors are regulated by lipids (PPARγ), bile acids (FXR), or free fatty acids (SREBP2).

Altered chemerin expression may be of relevance in the context of various pathological conditions like obesity, cancer, and inflammation^6,21,22^. Therefore, developing a better understanding of mechanisms underlying constitutive and regulated chemerin expression is of particular importance. In the present study, we demonstrate that the constitutive expression of chemerin is controlled by the DNA methylation of *RARRES2*, while the proximal region of the gene promoter is the key regulator of transcription. Moreover, using various experimental approaches we show that acute-phase cytokines, interleukin 1b (IL-1β) and oncostatin M (OSM), regulate chemerin expression in mouse primary adipocytes but not in hepatocytes, both in vitro and in vivo. As such, we provide novel insights into the mechanisms and factors affecting constitutive and regulated chemerin expression.

## Results

### Chemerin is constitutively expressed in liver and adipose tissue but not in B-cells

**Figure 1:**
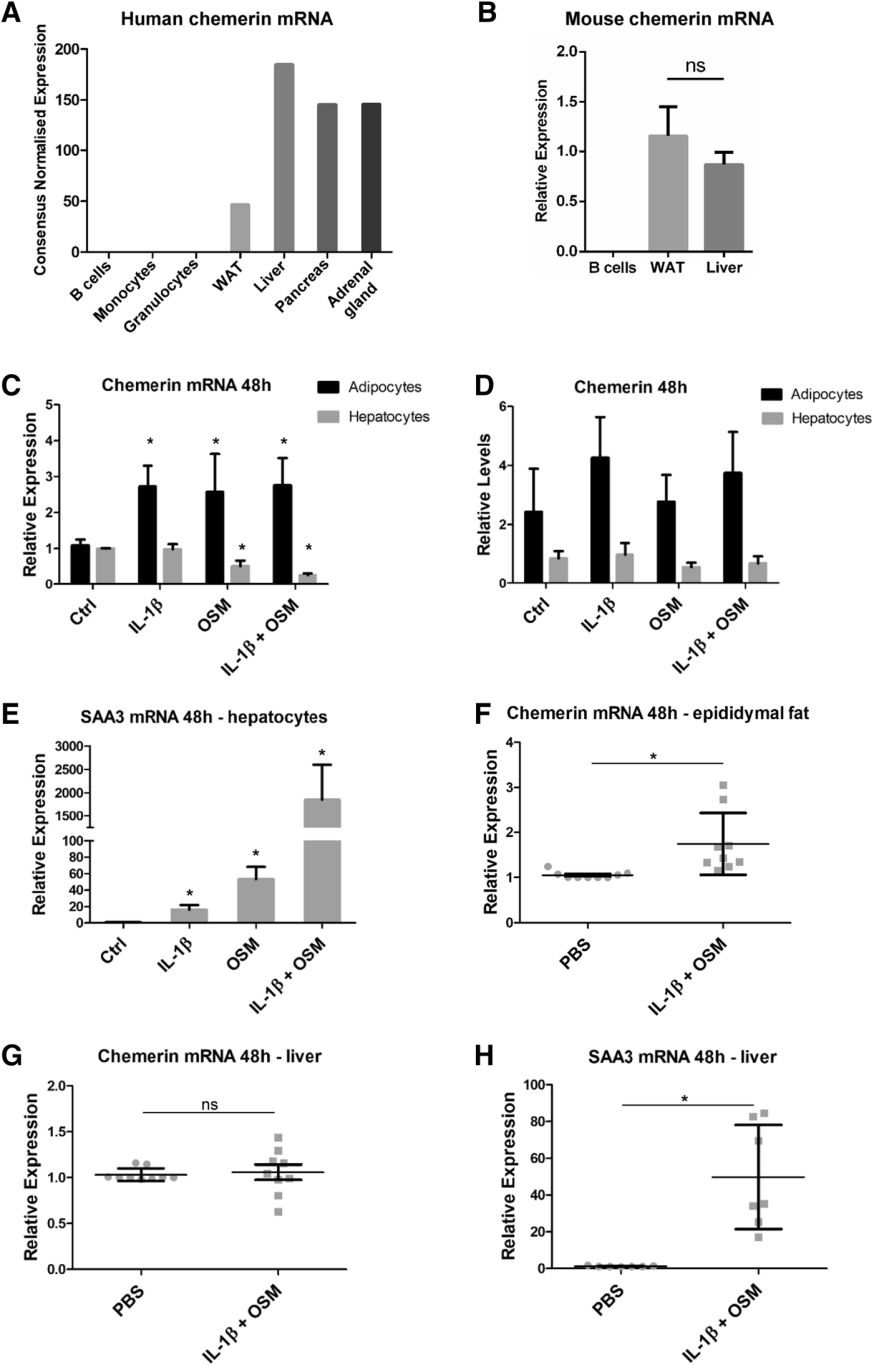
Chemerin is constitutively expressed in the liver and WAT. Acute-phase cytokines upregulate chemerin expression in the adipocytes of WAT but not in hepatocytes. The Human Protein Atlas was used to compare human chemerin mRNA levels across multiple tissues and cells (**A**). B-cells, WAT, and liver tissue were chosen for further studies. Afterward, lymph nodes, liver tissue, and eWAT depots were excised from C57Bl6 mice and subjected to RT-QPCR analysis or isolation of B-cells, primary hepatocytes, or the SVF of eWAT. Relative chemerin mRNA levels across murine B-cells, liver tissue, and WAT are shown (**B**). SVF cells were differentiated to obtain a mature adipocyte cell culture. Then, the cells were treated with IL-1β (10 ng/mL), OSM (50 ng/mL), or a combination for 48 h. The levels of chemerin (**C**) and SAA3 (**E**) mRNA were determined using RT-QPCR. The relative expression of stimulated cells over the control is shown. Levels of secreted chemerin were determined in parallel in conditioned media by ELISA (**D**). Data are presented as the mean ± SD of at least three independent experiments. Statistical significance between the control and treated cells is shown by an asterisk; *p < 0.05 by ANOVA followed by a Bonferroni post-hoc test. In vivo, IL-1β and OSM were injected intraperitoneally at doses of 10 μg/kg BW and 160 μg/kg BW, respectively. After 48 h, liver tissue and eWAT were isolated and subjected to RT-QPCR analysis. The levels of chemerin mRNA in eWAT (**F**) or liver tissue (**G**) and SAA3 (**H**) were determined. Data are presented as the mean ± SD of at least three independent experiments. Statistical significance between the control (PBS) and the cytokine-treated animals is indicated by an asterisk; *p < 0.05 by the two-tailed Student’s t-test. All experiments were repeated at least three times.

To study the constitutive and regulated expression of *RARRES2* we queried the Human Protein Atlas^16^ to identify cells and tissues having, on average, high mRNA levels of human chemerin or cells with very low or undetectable transcript levels. The liver, adrenal gland, pancreas, and white adipose tissue (WAT) show high chemerin mRNA levels but the transcript is not detectable in B lymphocytes, monocytes or granulocytes (Fig. 1A). Liver, WAT and B-cells were chosen for further analyses. Consistent with human data, RT-QPCR demonstrated that *RARRES2* was constitutively expressed in liver and WAT tissue but not in B-cells in mice (Fig. 1B).

### IL-1β and OSM stimulation upregulates ***RARRES***2 expression in murine adipocytes but not hepatocytes

Next, we questioned how chemerin expression can be regulated in cells with apparent constitutive expression of *RARRES2*. WAT and the liver have been reported by multiple studies to be key sites of chemerin production^1^. Both organs contribute to fatty acid metabolism, respond to numerous cytokines and are involved in the pathogenesis and pathophysiology of obesity^23^ which is linked to elevated systemic levels of chemerin^22^. Among the potential regulators of systemic chemerin levels are the acute-phase mediators OSM and IL-1β, which, as we showed previously, regulate chemerin levels in human epidermis-like cultures^2^. Moreover, liver and fat tissue are known to be responsive to both OSM and IL-1β^24–27^, suggesting that chemerin expression will be also regulated by these mediators in both adipocytes and hepatocytes. Treatment of epididymal white adipose tissue (eWAT)-derived mouse adipocytes with OSM and/or IL-1β resulted in statistically significant and comparable upregulation of *RARRES2* mRNA by each stimulus (Fig. 1C). In parallel, secreted chemerin protein levels tended to be higher after 48-h of stimulation with the cytokines as compared with the control (Fig. 1D). In contrast with the adipocytes, downregulation of *RARRES2* mRNA was detected in hepatocytes in response to OSM or OSM + IL-1β but not IL-1β alone (Fig. 1C). Likewise, cytokines did not affect the chemerin protein levels in hepatocyte-conditioned media (Fig. 1D). These in vitro results were corroborated by in vivo findings. *RARRES2* mRNA was upregulated only in eWAT (Fig. 1F) but not in the liver (Fig. 1G) after in vivo IL-1β + OSM administration. We confirmed that primary hepatocytes and mouse liver tissue responded to stimulation with IL-1β and OSM, since *SAA3* mRNA levels, encoding an acute-phase protein, was markedly elevated (Fig. 1E,H). Together, these results suggest that the effect of cytokines that results in the upregulation of chemerin expression is specific to fat tissue.

### Adipocytes are key cells expressing chemerin after stimulation with acute-phase cytokines

**Figure 2:**
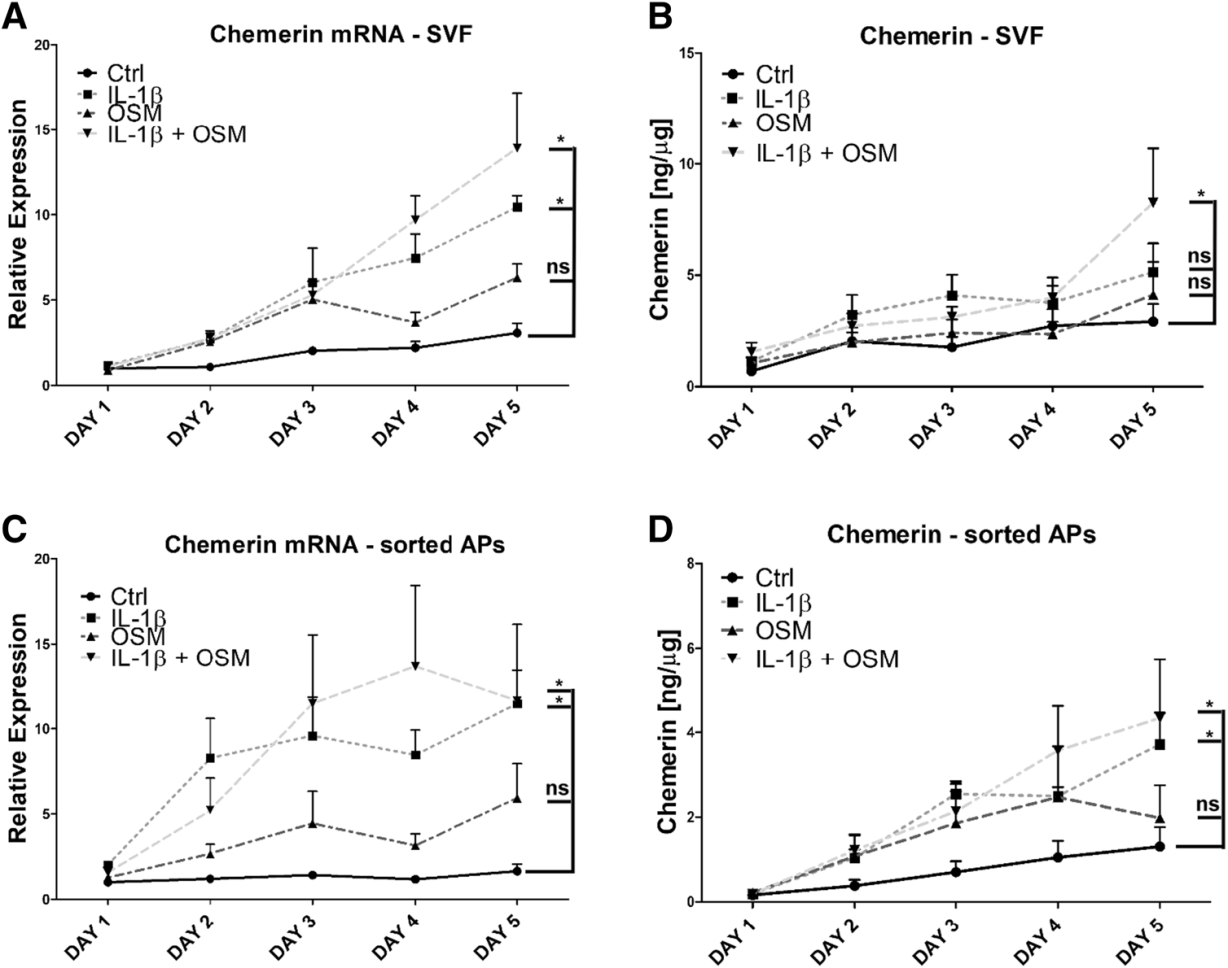
Adipocytes are key cells expressing chemerin following stimulation with IL-1β + OSM. eWAT or SCA-1 + APs were isolated. The cells were differentiated to obtain a mature adipocyte cell culture. Then, the cells were treated with IL-1β (10 ng/mL), OSM (50 ng/mL), or a combination for up to five days. The levels of chemerin mRNA (**A**, **C**) were determined using RT-QPCR. The relative expression of stimulated cells over the control is shown. Levels of secreted chemerin were determined in parallel in conditioned media by ELISA (**B**, **D**). Data are presented as the mean ± SEM of at least three independent experiments. Statistical significance between the control and the treated cells is indicated by an asterisk; *p < 0.05 by analysis of covariance followed by a Bonferroni post-hoc test.

The stromal vascular fraction (SVF) of adipose tissue consists of a heterogeneous population of cells that includes adipocyte precursors, hematopoietic stem cells, endothelial cells, fibroblasts, and immune cells^28^. Therefore, we next asked whether adipocytes are the main cells that express chemerin in response to acute-phase cytokines. Indeed, we found a similar chemerin expression pattern and levels in both eWAT-derived adipocytes and in adipocytes derived from sorted SCA-1^+^ adipogenic progenitors (Fig. 2A–D). Chemerin transcript levels continued to rise throughout a five-day time course. Stimulation with IL-1β and IL-1β + OSM yielded the highest mRNA levels (characterized by an approximately 12- to 13-fold increase relative to the one-day control) in both types of adipocyte cell culture (Fig. 2A,C). However, statistically significant upregulation of chemerin protein levels was observed only in the eWAT derived adipocytes treated by IL-1β + OSM or in the cell culture of sorted adipogenic precursors following IL-1β and IL-1β + OSM stimulation. Accordingly, we concluded that IL-1β and OSM regulate chemerin expression primarily in adipocytes. Chemerin protein production was highest in response to IL-1β + OSM, suggesting the existence of additive effects.

### DNA methylation affects constitutive *RARRES2* expression

We next investigated DNA methylation of the *RARRES2* promoter region as a possible mechanism underlying differential^29^ constitutive and regulated chemerin expression in various cell types. We studied DNA fragments ranged ranging in location from − 735 to + 258 bp of the *RARRES2* gene because promoter sequences are typically defined as being 100 to 1,000 bp upstream of the transcription start site and 100 bp downstream of the transcription start site^30^. Moreover, this DNA region contains all the binding sites of previously characterized transcription factors (TFs) of *RARRES2*^18–20^.

**Figure 3:**
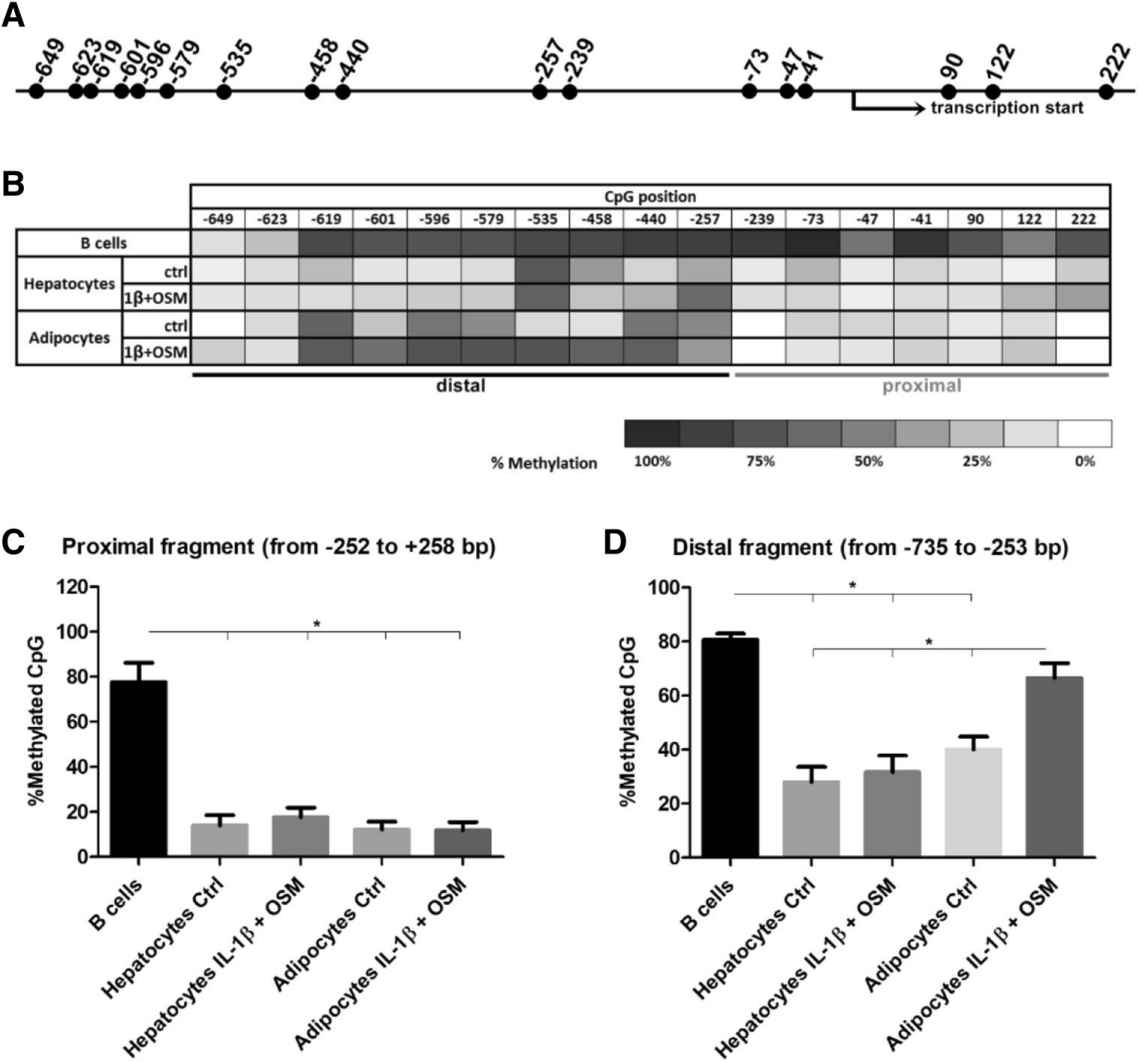
Chemerin transcription is linked to the DNA methylation status of the *RARRES2* promoter region from − 735 to + 258 bp. Primary mouse hepatocytes and mature adipocytes derived from sorted APs were treated with IL-1β (10 ng/mL) and OSM (50 ng/mL) for 48 h. B-cells, which do not produce chemerin, were adopted as a control. The DNA methylation status of the *RARRES2* promoter sequence was analyzed by bisulfite sequencing. The localization of CpG sites (**A**) and the DNA methylation levels of each CpG site from − 735 to + 258 bp of the *RARRES2* promoter are shown (**B**). The differences in the average methylation level of the proximal (**C**) and distal (**D**) parts of the promoter were analyzed using the nonparametric Kruskal–Wallis test; *p < 0.05. Data are presented as the mean ± SEM of at least three independent experiments.

Using bisulfite sequencing we determined the methylation status of the murine *RARRES2* gene in B-cells that do not produce chemerin, as well as that in unstimulated and cytokine-treated adipocytes and hepatocytes. We identified 17 CpG sites located within − 735/+ 258 bp of *RARRES2* (Fig. 3A). However, a computational analysis of this sequence did not identify any CpG island (data not shown). The chemerin promoter was found to be highly methylated in B lymphocytes. In contrast, our results suggested a much lower methylation status of the chemerin promoter in unstimulated adipocytes and hepatocytes relative to in B-cells (Fig. 3B). Interestingly, the upregulation of chemerin expression after stimulation of adipocytes with IL-1β and OSM is correlated with the statistically significant increase in the average methylation level of the *RARRES2* gene promoter within − 735/− 253 bp (Fig. 3C) but not − 252/+ 258 bp (Fig. 3D). However, the methylation pattern of the chemerin promoter was not altered in the cytokine-treated but chemerin-unresponsive primary cultures of mouse hepatocytes. We concluded that DNA methylation plays a role in controlling the constitutive expression of chemerin and affects cytokine-regulated expression in adipocytes.

### The proximal part of the *RARRES2* promoter is a key regulator of transcription

**Figure 4:**
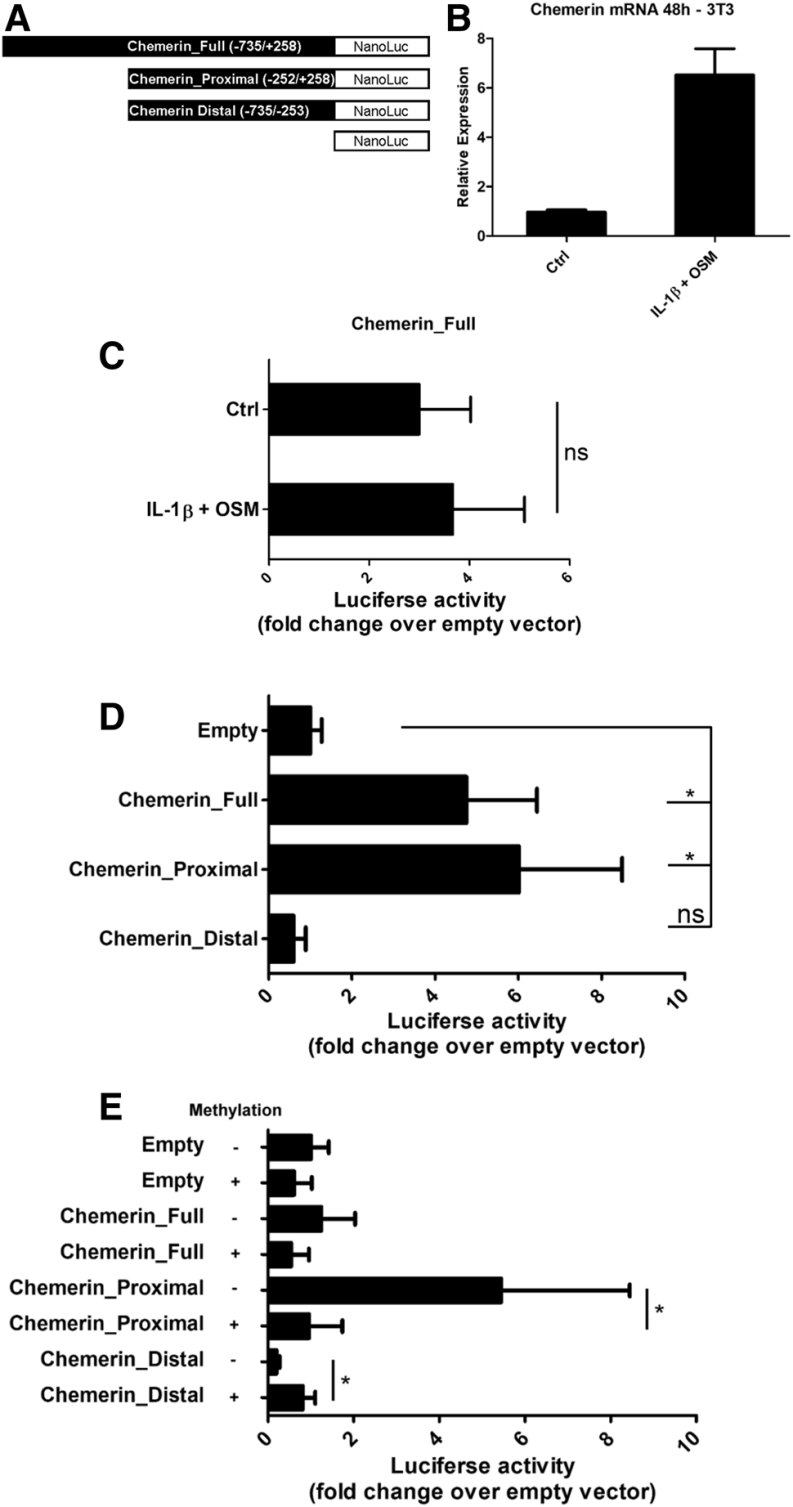
The proximal part of the chemerin promoter is a key regulator of transcription. The murine *RARRES2* promoter regions (from − 735 to + 258 bp) were cloned into the pNL1.1[Nluc]-Basic vector (**A**). 3T3-L1 adipocyte precursors were used for transient transfections since these cells respond to IL-1β + OSM stimulation similarly to the primary adipocytes (**B**). 3T3-L1 cells were transfected with Chemerin_Full vector (**C**) or different chemerin promoter constructs (**D**, **E**) and eventually stimulated with cytokines. The results are expressed as the fold-change in relative luciferase units relative to the empty vector. Statistical significance is indicated by an asterisk; *p < 0.05 by ANOVA followed by a Bonferroni post-hoc test. The Mann–Whitney test was used to analyze statistical differences between methylated/mock methylated vectors. Data are presented as the mean ± SD of at least three independent experiments.

To test how cytokines or DNA methylation can regulate the transcription of *RARRES2*, we established the following three reporter constructs containing different portions of chemerin promoter sequences: Chemerin_Full (position − 735/+ 258 bp relative to the transcription start site), Chemerin_Proximal (position − 252/+ 258 bp), and Chemerin_Distal (position − 735/− 253 bp) (Fig. 4A). The promoter was divided into two parts based on the methylation levels of *RARRES2* (Fig. 3B). The average percentage methylation levels for the proximal region was less than 14% in both unstimulated adipocytes and hepatocytes whereas the distal part of the promoter showed greater average methylation, varying from 28% (hepatocytes) to 40% (adipocytes). We used 3T3-L1 adipocyte precursors for transient transfection since these cells produce chemerin and respond to cytokine stimulation in a manner similar to that of differentiated adipocytes (Fig. 4B).

First, we considered whether the Chemerin_Full construct, which covers all previously characterized TF binding sites^18–20^, responds to IL-1β + OSM stimulation. However, cytokine treatment did not reach statistical significance relative to in the untreated controls (Fig. 4C). To investigate whether inflammation-responsive TFs may bind to − 735/+ 258 bp of the *RARRES2* promoter sequence in murine adipocytes and hepatocytes, we queried ChIP-Atlas^31^, a resource that gathers publicly available results from chromatin immunoprecipitation-sequencing experiments. The results showed that inflammation-responsive TFs, including NFκB, AP-1, SAF-1, c-Jun/c-Fos, and STATs, do not bind to the − 735/+ 258 bp of the *RARRES2* promoter sequence either in adipocytes or in hepatocytes (Table 1).

**Table 1:** TFs that may bind the *RARRES2* promoter region located from − 735 to + 258 bp in murine adipocytes and hepatocytes.

Transcription factor	Chromosome	Start	End	SRX ID	q-value	− 10 × log_10_[q]
**Adipocytes**						
Nr3c1	chr6	48,522,605	48,522,985	SRX821805	1.00E−100	1,000
Pparg	chr6	48,522,627	48,522,940	SRX821796	1.00E−100	1,000
Nr3c1	chr6	48,522,608	48,522,979	SRX821802	5.01E−85	843
Pparg	chr6	48,522,641	48,522,924	SRX821794	7.94E−81	801
Pparg	chr6	48,522,639	48,522,919	SRX821793	7.94E−74	731
Pparg	chr6	48,522,600	48,522,886	SRX821792	7.94E−72	711
**Liver**						
Ppara	chr6	48,522,633	48,522,867	SRX5142529	1.00E−100	1,000
Rxra	chr6	48,522,578	48,522,927	SRX020176	1.00E−100	1,000
Ncor1	chr6	48,522,654	48,522,891	SRX5028170	1.00E−100	1,000
Ncor1	chr6	48,522,651	48,522,880	SRX5028173	1.00E−100	1,000
Ncor1	chr6	48,522,646	48,522,866	SRX5028172	1.00E−100	1,000
Ncor1	chr6	48,522,662	48,522,870	SRX5028171	1.00E−100	1,000
Ppara	chr6	48,522,629	48,522,872	SRX5142530	1.00E−100	1,000
Ppara	chr6	48,522,627	48,522,900	SRX5142531	1.00E−100	1,000
Rxra	chr6	48,522,625	48,522,927	SRX020179	1.00E−100	1,000
Rxra	chr6	48,522,604	48,522,939	SRX020180	1.00E−100	1,000
Ncor1	chr6	48,522,664	48,522,868	SRX5028169	1.00E−100	1,000
Rxra	chr6	48,522,585	48,522,946	SRX020175	1.00E−100	1,000
Ncor1	chr6	48,522,657	48,522,869	SRX5028168	1.00E−100	1,000
Hnf4a	chr6	48,522,552	48,522,951	SRX547094	1.00E−100	1,000
Hnf4a	chr6	48,522,508	48,522,984	SRX2375607	1.00E−100	1,000
Hnf4a	chr6	48,522,561	48,522,954	SRX547095	1.00E−100	1,000
Hnf4a	chr6	48,522,517	48,522,959	SRX2375608	1.00E−100	1,000
Ppara	chr6	48,522,638	48,522,866	SRX5028181	7.94E−96	951
Ppara	chr6	48,522,643	48,522,842	SRX5142527	1.26E−95	949
Rxra	chr6	48,522,620	48,522,928	SRX4949978	1.58E−94	938
Ppara	chr6	48,522,637	48,522,885	SRX5028184	3.16E−92	915
Cebpb	chr6	48,522,643	48,522,907	SRX661416	1.58E−86	858
Ppara	chr6	48,522,640	48,522,853	SRX5028180	1.58E−82	818
Ppara	chr6	48,522,646	48,522,888	SRX5028185	7.94E−80	791
Rxra	chr6	48,522,524	48,522,942	SRX6658445	5.01E−71	703

ChIP-Atlas^31^ raw data linked to the selected records were downloaded and submitted to CRUNCH online tool^59^ to identify proteins bound to the *RARRES2* genomic loci with the binding score value of − 10 × log10[q] > 700.

To determine which part of the *RARRES2* promoter region is linked with constitutive chemerin expression, 3T3-L1 cells were transfected with the Chemerin_Full, Chemerin_Proximal, and Chemerin_Distal constructs, respectively. Chemerin_Proximal and Chemerin_Full showed the highest promoter activity as determined by luciferase assay (Fig. 4D). The Chemerin_Distal construct had similar promoter activity in comparison with the empty vector.

To investigate the role of DNA methylation in the regulation of chemerin expression, we excised the promoter sequence from pNL1.1[Nluc] constructs with restriction enzymes, treated the eluted DNA with SssI methylase or left it untreated, and relegated the methylated and unmethylated DNA inserts into the parent vector. Promoter activity of the methylated Chemerin_Proximal construct was five times lower than that of the unmethylated construct (Fig. 4E). In line with the results of bisulfite sequencing (Fig. 3), promoter activity of the methylated Chemerin_Distal construct was higher than that of the unmethylated construct.

Together, these data suggest that the DNA sequence of the *RARRES2* promoter region located from − 252 to + 258 bp is critical for constitutive chemerin expression and that its methylation suppresses the transcription. Interestingly, the distal part of the chemerin promoter (position − 735/− 253 bp) shows minimal activity which is increased when DNA is methylated.

## Discussion

Control of gene expression plays a key role in a variety of physiological and pathophysiological processes, ranging from cell differentiation, cellular stress responses, and immunity, to tissue homeostatis. Many mechanisms contribute to the regulation of gene expression to ensure coordinated cellular behaviors and fate decisions. This includes modifications of DNA (e.g. DNA methylation), binding of TFs to a gene promoter, alternative splicing, miRNAs, and many others^32^. To date, the molecular mechanisms controlling constitutive and regulated chemerin expression are still poorly understood^1^. Further, the functional significance of DNA methylation in the regulation of *RARRES2* transcription has not yet been elucidated.

Here, we determined, for the first time, that CpG sites located between − 735 and + 258 bp of the *RARRES2* promoter are highly methylated in B-cells but not in hepatocytes and adipocytes. This suggests that DNA methylation controls constitutive chemerin expression in cells of different origins. B-cells do not secrete chemerin; meanwhile, hepatocytes and adipocytes are known to express high levels of chemerin and to release the chemerin protein^1^. This constitutive expression controls tissue homeostasis by affecting physiological processes like immune cell migration, angiogenesis, adipogenesis, and energy metabolism^33^. Epigenetic regulation by CpG methylation in the promoter region is often associated with both tissue-specific and heterogeneous expression of genes^29^. DNA methylation can cause transcriptional silencing of genes by inhibiting the binding of TFs to regulatory sequences^34^. Even the methylation status of a single CpG locus can modulate gene expression^35,36^. However, we have also observed variations in the DNA methylation pattern between the proximal (− 252/+ 258 bp) and distal (− 735/− 253 bp) parts of the *RARRES2* promoter region in adipocytes and hepatocytes. Therefore, we divided the chemerin promoter into two parts to study activity. Luciferase assays revealed that the proximal part of the chemerin promoter is a key regulator of constitutive chemerin expression. Methylation of the CpG sites located within this region diminished luciferase activity. Finally, the distal part of the chemerin promoter is poorly characterized and did not show any important activity in luciferase assays. Taken together, the DNA methylation of the *RARRES2* promoter region located from − 252 to + 258 bp regulates the constitutive expression of chemerin in a cell-type dependent manner.

The expression of chemerin may be also regulated by various mediators including IL-1β and OSM acute-phase cytokines. Both IL-1β, and OSM, are essential mediators of adaptive and innate immune responses^37,38^, and both have potential roles in the pathology of psoriasis^39,40^, rheumatoid arthritis^41^, cancer^42^, and obesity^43^, which are diseases with postulated chemerin involvement^6,21, 22,44^. We previously showed that IL-1β and OSM upregulated chemerin expression in human skin cultures^2^. Here, our research revealed that a combination of both cytokines considerably increased *RARRES2* mRNA and protein levels in cell cultures of murine eWAT-derived adipocytes and differentiated SCA1 + APs but not in primary hepatocytes. This result is in agreement with those reported by Kralisch et al.^45^ who found that IL-1β increased chemerin expression in 3T3-L1 adipocytes. Also, these in vitro results were corroborated by in vivo findings: *RARRES2* mRNA was elevated in eWAT but not in the liver and chemerin was up-regulated, on average, by 1.5- to 2.0-fold in eWAT. Although this increase in chemerin levels was not robust, eWAT-mediated local production and/or activation of chemerin may play an important role in maintaining tissue homeostasis and should be considered in addition to systemic levels of circulating chemerin^1,46,47^. In contrast with adipocytes, IL-1β and OSM did not affect chemerin expression in hepatocytes. The existence of differential regulation of chemerin expression in the liver and adipose tissue in response to other mediators has also been reported by other research groups^17,48^. So far, only FFAs and GW4064, a synthetic FXR agonist, have been shown to influence chemerin expression in hepatocytes^19^.

We also asked how the *RARRES2* promoter region affects the regulated expression of chemerin. Analysis of the Chemerin_Full construct did not reveal any statistically significant degree of activation over the unstimulated control when transfected cells were stimulated with IL-1β and OSM. The Chemerin_Full vector, which covers − 735 to + 258 bp region of the murine *RARRES2* gene, contains all previously characterized functional TF binding sites: including the PPAR response element^20^, FXR response element^19^, and SREBP2 binding site^18^. Acute phase cytokines can impact the expression of inflammatory-responsive genes by a variety of TFs including NFκB, AP-1, c-Jun/c-Fos, IRF1 or STAT1^49–52^. However, our search of the ChIP-Atlas database^31^ did not reveal that any of the listed TFs were able to bond to the − 735/+ 258 bp of the *RARRES2* promoter region either in adipocytes or hepatocytes. This may indicate that inflammation-responsive TFs binding sites are located outside of the investigated promoter region or that other mechanisms and/or cis-regulatory elements affect chemerin expression.

Interestingly, the upregulation of chemerin expression after stimulation of adipocytes with cytokines correlated with a statistically significant increase in the average methylation level of the distal region of the *RARRES2* promoter. The methylation pattern was not altered in cytokine-treated but chemerin-unresponsive primary cultures of mouse hepatocytes. Methylation of the Chemerin_Distal construct increased luciferase activity when compared with the unmethylated vector, but such was still far below the activity of the unmethylated proximal region. DNA methylation is typically associated with gene silencing although CpG methylation of the DNA and, like methylation of the CRE sequence, may enhance the DNA binding of TFs^53^.

In summary, our studies reveal novel insights into the mechanisms and factors regulating chemerin expression and secretion. For the first time, we show that DNA methylation may control the constitutive expression of *RARRES2,* and that the proximal region of the gene promoter is the key regulator. Acute-phase cytokines affect chemerin expression in a cell-type dependent manner, both in vitro and in vivo. The investigated *RARRES2* promoter region was unresponsive to acute-phase cytokine stimulation. These findings provide a basis for further investigations of the regulation of chemerin transcription.

## Methods

### Materials

If not stated differently, all chemicals were purchased from Sigma-Aldrich (St. Louis, MO, USA). Roswell Park Memorial Institute (RPMI)-1640 medium was obtained from Biowest. Dulbecco's Modified Eagle medium (DMEM), DMEM:F12 medium, and phosphate-buffered saline (PBS) buffer were purchased from PAN Biotech (Aidenbach, Germany). FBS was purchased from Gibco Laboratories (Gaithersburg, MD, USA). Bovine serum albumin (BSA), ethylenediaminetetraacetic acid (EDTA), and trypan blue were purchased from BioShop. Collagenase D was obtained from Roche Holding AG (Basel, Switzerland). Mouse recombinant IL-1β and OSM were purchased from R&D Systems (Minneapolis, MN, USA). Fc block [rat–anti-mouse cluster of differentiation (CD)16/32, #101310], biotin-conjugated rat–anti-mouse CD45 (#103104), biotin-conjugated rat–anti-mouse CD31 (#102404), and PE-conjugated rat–anti-mouse Ly-6A/E (#108108) antibodies were purchased from Biolegend (San Diego, CA, USA). Lineage selection columns, lineage depletion columns, anti-biotin, and anti-PE magnetic beads were purchased from Miltenyi Biotec (Bergisch Gladbach, Germany). PCR primers were obtained from Genomed (Leesburg, FL, USA).

### Animal studies

Male eight- to 12-week-old C57BL/6 mice were used for these investigations. The mice were maintained under specific pathogen-free conditions at the Faculty of Biochemistry, Biophysics, and Biotechnology of Jagiellonian University animal care facility. IL-1β and OSM were injected intraperitoneally at doses of 10 μg/kg BW and 160 μg/kg BW, respectively. After 48 h, the liver and eWAT were isolated and subjected to RT-QPCR analysis. All experimental procedures were approved by the First Local Ethical Committee on Animal Testing at the Jagiellonian University in Krakow, Poland (permit no. 41/2014), in accordance with the Guidelines for Animal Care and Treatment of the European Community. The mice were sacrificed by an overdose of anesthesia (a mixture of ketamine and xylasine), followed by cervical dislocation.

### Primary hepatocytes isolation and culture

Primary hepatocytes were isolated from C57BL6 mice with a modified two-step perfusion method according to the protocol described by Seglen^54^. Briefly, the mice were anesthetized with ketamine (100 mg/kg) and xylazine (10 mg/kg) intraperitoneally and the abdomen was opened under sterile conditions. Following cannulation of the portal vein, the liver was perfused with solution I [100 µM of EGTA in Krebs–Ringer (K–R) buffer], followed by solution II (1 mg/mL of collagenase D in K-R buffer supplemented with 150 µM of CaCl_2_). Then, the liver was dissected, passed through a 100-µm cell strainer, and centrifuged (60 g, five minutes, 10 °C). The isolated hepatocytes were suspended in DMEM:F12 medium supplemented with 10% FBS, 50 µg/mL of gentamycin, 6 ng/mL of insulin, and 400 ng/mL of dexamethasone. Viable cells were counted using trypan blue staining and seeded on a collagen-coated plate at a density of 1 × 10^5^ cells/cm^2^. Cells were cultured at 37 °C in the presence of 5% CO_2_ for four hours. Afterward, plated cells were washed with DMEM:F12 medium and stimulated with IL-1β (10 ng/mL) and OSM (50 ng/mL) for 48 h. At the time of harvest, cell culture media supernatants were collected and RNA lysis buffer (Fenozol Plus; A&A Biotechnology, Gdynia, Poland) or radioimmunoprecipitation assay buffer containing protease inhibitors (Roche Holding AG, Basel, Switzerland) were added to the specified wells. Collected samples were subjected to RT-QPCR or enzyme-linked immunosorbent assay (ELISA) analysis.

### Isolation and culture of adipose tissue-derived stromal vascular fraction (SVF)

eWAT depots were isolated from 8- to 10-week-old male C57BL6 mice, minced, and digested with collagenase D (3.5 mg/mL) in K–R buffer supplemented with 2% BSA and 150 μM of CaCl_2_ for one hour in 37 °C water bath with shaking every five minutes. Digested tissue was then centrifuged (280 g, 10 min, 15 °C), washed, filtered through 100 μm of cell strainer, and centrifuged. Then, the pellet was suspended in red blood cell lysis buffer (155 mM of NH_4_Cl, 12 mM of NaHCO_3_, 0.1 mM of EDTA) for three minutes at room temperature, centrifuged, and suspended in DMEM:F12 medium (20% FBS, gentamycin 50 μg/mL). Isolated SVF cells were then seeded at a density of 9 × 10^4^ cells/cm^2^ on a culture plate. Attached cells were washed and replenished with fresh medium after 24 h to discard unattached dead cells or immune cells and, by the third day, more than 90% of cells displayed typical fibroblastic morphology. Then, the SVF culture was subjected to adipocyte differentiation.

### Isolation and culture of adipogenic progenitors

Adipogenic progenitors (APs) were isolated from C57BL6 mice according to the protocol described by Lee et al.^55^. eWAT was digested, filtered, and washed as described above. APs were then isolated first using negative selection of CD45 and CD31 and then positive selection for SCA-1. Briefly, SVF cells were suspended in MACS buffer (0.5% BSA, 2 mM of EDTA, 50 µg/mL of gentamycin) and preincubated with Fc block [rat–anti-mouse CD16/32 monoclonal antibody (mAb), 45 µg/mL], which was followed by incubation with biotin-conjugated antibodies against CD45 (rat–anti-mouse CD45 mAb) and CD31 (rat–anti-mouse CD31 mAb), both 7.5 μg/mL. Then, SVF cells were incubated with streptavidin-conjugated magnetic beads, washed, and passed over a lineage depletion column to exclude endothelial cells and leukocytes. The flow-through was collected, washed, and incubated with PE-conjugated anti-SCA-1 (rat–anti-mouse Ly-6A/E mAb, 4.8 μg/mL), followed by anti–PE-conjugated magnetic beads. Cells were then washed and passed over a lineage selection column. Labeled cells were collected, suspended, in DMEM:F12 medium (20% FBS, gentamycin 50 μg/mL), counted, seeded on a cell culture flask, and expanded. Then, the cells were replated at a density of 2.5 × 10^4^ cells/cm^2^ and subjected to adipocyte differentiation in adipocyte maintenance medium.

### Adipocyte differentiation

Differentiation was induced when SVF cells or APs reached 90% confluence. Cells were switched to adipocyte differentiation medium (DMEM:F12, 8% FBS, 8 µg/mL of biotin, 50 µg/mL of gentamycin, 1.15 µg/mL of insulin, 80 µg/mL of IBMX, 1.5 µg/mL of troglitasone, 0.4 µg/mL of dexamethasone) for 4 days. Subsequently, adipocyte differentiation medium was changed to adipocyte maintenance medium (DMEM:F12, 8% FBS, 8 µg/mL of biotin, 50 µg/mL of gentamycin, 1.15 of µg/mL insulin) for another seven days. Subsequently, the cells were stimulated with cytokines as described above.

### Isolation of B lymphocytes

Popliteal and axillary lymph nodes and the spleen were dissected and pressed through 40-µm mesh. Cells were washed with RPMI-1640, centrifuged (300 g, 6 min, 4 °C), and the pellet was suspended in red blood cell lysis buffer for five minutes at room temperature. Cells were centrifuged and suspended in MACS buffer (3% FBS, 10 mM of EDTA in PBS). B-cell magnetic sorting was conducted using the MagniSort Negative Selection Protocol II (MagniSort Mouse B-cell Enrichment Kit; Affymetrix, Santa Clara, CA, USA). Negatively selected cells were suspended in PBS and DNA extraction was performed.

### 3T3-L1 cell line culture and cytokine stimulation

The primary mouse preadipocyte cell line 3T3-L1 was purchased from the American Type Culture Collection. 3T3-L1 cells were grown in DMEM medium supplemented with 10% FBS and gentamycin (50 µg/mL). Cells were seeded at a density of 8 × 10^3^ cells/cm^2^ on a culture plate. After 24 h, the medium was changed and cells were stimulated with IL-1β (10 ng/mL) and OSM (50 ng/mL) for 48 h. At the time of harvesting, cell culture media was removed and RNA lysis buffer was added. Collected samples were subjected to RT-QPCR analysis.

### RT-QPCR

Total RNA was extracted with the Total RNA Zol-Out Kit (A&A Biotechnology, Gdynia, Poland) and converted to complementary DNA using NxGen M-MulV reverse transcriptase (Lucigen Corporation, Middleton, WI, USA) with random primers (Promega Corporation, Madison, WI, USA). Real-time PCR was performed on the CFX96 thermocycler (Bio-Rad Laboratories, Hercules, CA, USA) using SYBR Green I containing universal PCR master mix (A&A Biotechnology, Gdynia, Poland) and the following primers specific to mice: chemerin (5′-CTTCTCCCGTTTGGTTTGATTG, 5′-TACAGGTGGCTCTGGAGGAGTTC), SAA3 (5′-ACAGCCAAAGATGGGTCCAGTTCA, 5′-ATCGCTGATGACTTTAGCAGCCCA), cyclophilin A (5′-AGCATACAGGTCCTGGCATCTTGT, 5′-CAAAGACCACATGCTTGCCATCCA) and β-actin (5′-CCTTCTTGGGTATGGAATCCTG, 5′-TGGCATAGAGGTCTTTACGGA). The Microsoft Excel–based (Microsoft Corporation, Redmond, WA, USA) application Best-Keeper was used to analyze the expression stabilities of commonly used reference genes^56^. Based on this analysis, murine cyclophylin A and β-actin were selected as housekeeping genes for normalizing RNA expression in RT-QPCR. Relative gene expression normalized to the geometric mean of these housekeeping genes was calculated using the 2^−ΔΔCT^ method^57^.

### ELISA

Chemerin levels in cell culture supernatants were quantified by mouse-specific ELISA. MaxiSorp Nunc-Immuno Module (Thermo Fisher Scientific, Waltham, MA, USA) strips were coated with rat–anti-mouse mAb (MAB23251; R&D Systems, Minneapolis, MN, USA) in Tris-buffered saline (50 mM of Tris–HCl with pH of 9.5, 150 mM of NaCl). The plates were then washed with PBS containing 0.1% Tween 20 and nonspecific protein-binding sites were blocked with 3% BSA in PBS. Mouse recombinant chemerin was used as a standard. Chemerin was detected using biotin-conjugated rat–anti-mouse chemerin mAb (BAM2325), followed by streptavidin–horseradish peroxidase (BD Biosciences, San Jose, CA, USA). The reaction was developed with TMB substrate (BD Biosciences, San Jose, CA, USA). The results were normalized to total protein content in the corresponding cell RIPA lysates (Quick Start Bradford Protein Assay; Bio-Rad Laboratories, Hercules, CA, USA). ELISA detected both the 163S and 157S chemerin.

### Bisulfite genomic DNA sequencing

Genomic DNA was extracted from B-cells, hepatocytes, or differentiated APs using the GeneJET Genomic DNA Purification Kit. Genomic DNA aliquots were then treated with sodium bisulfite using the EZ DNA Methylation-direct Kit (Zymo Research, Irvine, CA, USA). The targeted region of the RARRES2 promoter was amplified with PCR (ZymoTaq PreMix; Zymo Research, Irvine, CA, USA) using the following sets of primers specific to converted DNA: range − 717/+ 229 (5′-GAGAGATTGAGTTGGGGAAATGAG-3′ sense, 5′-CCCCAACCTCTTTCTAATACCTTA-3′ antisense, 62.0 °C), range − 246/+ 154 (5′-ATGATAAAGGAAAGGTAAAGGAAAGATTGGG-3′ sense, 5′-AAACAACTCCCTAACAATTATTCCCTCTCACC-3′ antisense, 53.0 °C), range − 459/− 160 (5′-GATGTTTGGTAGGTAGATGAAGGTAGTAGTTAGT-3′ sense, 5′-AACTACCATCAAAACAACTATCCCCAAC-3′ antisense, 58. 9 °C), range − 813/− 337 (5′-TAGGGAAAAGGTTTATTTGGTTAGTAGAGA-3′ sense, 5′-AAAAAAACTAAAACTCCTTCAATACCAAAA-3′ antisense, 50.2 °C). PCR products were then separated on 2% agarose gel and extracted with Gel-Out Concentrator (A&A Biotechnology, Gdynia, Poland). Purified DNA was cloned into pTZ57R/T vector (InsTAclone PCR Cloning Kit; Thermo Fisher Scientific, Waltham, MA, USA). After transformation and culturing of the competent bacteria (Top10 *Escherichia coli*; Thermo Fisher Scientific, Waltham, MA, USA) overnight on an LB/agar/ampicillin plate, at least eight colonies were randomly selected, plasmids were recovered using the GeneJET Plasmid Miniprep Kit, and the DNA was sequenced with M13 common sequencing primers. The results were analyzed using the QUMA online tool (RIKEN, Tsukuba, Japan).

### Plasmid construction and in vitro methylation

Livers from C57BL6 mice were dissected and genomic DNA was isolated using the GeneJET Genomic DNA Purification Kit. The *RARRES2* promoter sequence was amplified using Phusion high-fidelity DNA polymerase (Thermo Fisher Scientific, Waltham, MA, USA) and the following overlap extension PCR (OE-PCR) primers: 5′-CTCGAGGATATCAAGATCTGGCCTCGAAGCTTTCAGCTCCTCAGACAGGAA-3′ and 5′-GCTTTACCAACAGTACCGGATTGCCAAGTGGTACCTTGAAAATGATCAGGTTTGTT-3′ (− 735/+ 258; Chemerin_Full). The resulting PCR product was subcloned into the promoterless pNL1.1[Nluc] vector (#N1001; Promega Corporation, Madison, WI, USA) using OE-PCR as described by Bryksin and Matsumura^58^. Two additional constructs were then created using pNL1.1[Nluc]_Chemerin_Full as a template and the following OE-PCR primers: 5′-CTCGAGGATATCAAGATCTGGCCTCGATCTGTCAAAAAACGGCTCCCTCAAGTG-3′ and 5′-CACTTGAGGGAGCCGTTTTTTGACAGATCGAGGCCAGATCTTGATATCCTCGAG-3′ (− 252/+ 258; Chemerin_Proximal), 5′-GAAGATCACCTGGTCAAGCGGGGCTTGGCAATCCGGTACTGTTGGTAAAGC-3′ and 5′-GCTTTACCAACAGTACCGGATTGCCAAGCCCCGCTTGACCAGGTGATCTTC-3′ (− 735/− 253; Chemerin_Distal). The integrity and orientation of the inserts were confirmed by sequencing. Plasmids were amplified in *E. coli* and purified using Plasmid MIDI AX kit (A&A Biotechnology, Gdynia, Poland). For in vitro methylation studies, the constructs were digested with NcoI and BglII (New England BioLabs, Ipswitch, MA, USA) and inserts and vectors bands were separated and extracted from the agarose gel. Inserts were then incubated for one hour at 37 °C in the presence or absence of SssI methylase (New England BioLabs, Ipswitch, MA, USA). The efficiency of the methylation reaction was verified by resistance to cleavage using the methylation-sensitive restriction enzyme HpaII (New England BioLabs, Ipswitch, MA, USA). The methylated and mock-methylated chemerin promoter fragments were re-ligated into the parent vector, purified using Clean-Up Concentrator (A&A Biotechnology, Gdynia, Poland), and used for transfection.

### Transient transfection and luciferase assay

3T3-L1 cells were seeded at a density of 1.5 × 10^4^ cells in a 24-well culture plate and, 24 h later, cells were transfected using ViaFect transfection reagent (Promega Corporation, Madison, WI, USA). The total amount of DNA used for transfection per well was 0.8 μg, including approx. 0.5 μg of pNL1.1[Nluc] (equimolar concentrations of empty pNL1.1[Nluc], pNL1.1[Nluc]_Chemerin_Full, pNL1.1[Nluc]_Chemerin_Proximal, or pNL1.1[Nluc]_Chemerin_Distal) and 0.3 μg of the pGL4.54 [luc2/TK] vector (Promega Corporation, Madison, WI, USA) expressing the Firefly luciferase that was used as an internal control. Twenty-four hours after transfection, a portion of the cells was stimulated with IL-1β (10 ng/mL) and OSM (50 ng/mL) for 48 h. For methylation studies, cells were transfected with 0.5 μg of ligation reactions from the methylated/mock-methylated pNL1.1[Nluc]/chemerin promoter constructs and 0.3 μg of the pGL4.54 [luc2/TK] vector. The amount of DNA per well was equalized using mock plasmid DNA (pcDNA3.1; Promega). Forty-eight hours later, the cells were harvested by scraping into a passive lysis buffer (Promega Corporation, Madison, WI, USA). NanoLuc and Firefly activities in cell lysates were measured using the Nano-Glo Dual-Luciferase Reporter Assay System (Promega Corporation, Madison, WI, USA) according to the manufacturer’s protocols.

### Identification of TFs that bind to *RARRES2* promoter using ChIP-Seq

To investigate which TFs bind to *RARRES2* promoter sequence, we queried ChIP-Atlas^31^. Raw data linked to selected records were submitted to the CRUNCH online tool for peak verification^59^. We focused on the peaks with the q-value for statistical significance below 1/(10^70^).

### Statistical analysis

All data were analyzed using STATISTICA 13 (StatSoft, Tulsa, OK, USA), visualized with Prism (GraphPad Software, San Diego, CA, USA), and presented as mean ± standard deviation (SD) or mean ± standard error of the mean (SEM). The Student’s t-test was used for comparison between two groups. For multiple comparisons, either analysis of variance (ANOVA) with the Bonferroni post-hoc test or Kruskal–Wallis ANOVA was used. For experiments with repeated measures over time, analysis of covariance with the Bonferroni post-hoc test was used instead. Differences were considered statistically significant for p-values of less than 0.05.
